# Altered myocardial substrate metabolism is associated with myocardial dysfunction in early diabetic cardiomyopathy in rats: studies using positron emission tomography

**DOI:** 10.1186/1475-2840-8-39

**Published:** 2009-07-22

**Authors:** Charissa E van den Brom, Marc C Huisman, Ronald Vlasblom, Nicky M Boontje, Suzanne Duijst, Mark Lubberink, Carla FM Molthoff, Adriaan A Lammertsma, Jolanda van der Velden, Christa Boer, D Margriet Ouwens, Michaela Diamant

**Affiliations:** 1 Department of Endocrinology, Diabetes Centre, VU University Medical Centre, Amsterdam, The Netherlands; 2 Laboratory for Physiology, VU University Medical Centre, Amsterdam, The Netherlands; 3 Department of Nuclear Medicine & PET Research, VU University Medical Centre, Amsterdam, The Netherlands; 4 Department of Anaesthesiology, VU University Medical Centre, Amsterdam, The Netherlands; 5 Department of Molecular Cell Biology, LUMC, Leiden, The Netherlands

## Abstract

**Background:**

*In vitro *data suggest that changes in myocardial substrate metabolism may contribute to impaired myocardial function in diabetic cardiomyopathy (DCM). The purpose of the present study was to study in a rat model of early DCM, *in vivo *changes in myocardial substrate metabolism and their association with myocardial function.

**Methods:**

Zucker diabetic fatty (ZDF) and Zucker lean (ZL) rats underwent echocardiography followed by [^11^C]palmitate positron emission tomography (PET) under fasting, and [^18^F]-2-fluoro-2-deoxy-D-glucose PET under hyperinsulinaemic euglycaemic clamp conditions. Isolated cardiomyocytes were used to determine isometric force development.

**Results:**

PET data showed a 66% decrease in insulin-mediated myocardial glucose utilisation and a 41% increase in fatty acid (FA) oxidation in ZDF vs. ZL rats (both p < 0.05). Echocardiography showed diastolic and systolic dysfunction in ZDF vs. ZL rats, which was paralleled by a significantly decreased maximal force (68%) and maximal rate of force redevelopment (69%) of single cardiomyocytes. Myocardial functional changes were significantly associated with whole-body insulin sensitivity and decreased myocardial glucose utilisation. ZDF hearts showed a 68% decrease in *glucose transporter-4 *mRNA expression (p < 0.05), a 22% decrease in glucose transporter-4 protein expression (p = 0.10), unchanged levels of pyruvate dehydrogenase kinase-4 protein expression, a 57% decreased phosphorylation of AMP activated protein kinase α1/2 (p < 0.05) and a 2.4-fold increased abundance of the FA transporter CD36 to the sarcolemma (p < 0.01) vs. ZL hearts, which are compatible with changes in substrate metabolism. In ZDF vs. ZL hearts a 2.4-fold reduced insulin-mediated phosphorylation of Akt was found (p < 0.05).

**Conclusion:**

Using PET and echocardiography, we found increases in myocardial FA oxidation with a concomitant decrease of insulin-mediated myocardial glucose utilisation in early DCM. In addition, the latter was associated with impaired myocardial function. These *in vivo *data expand previous *in vitro *findings showing that early alterations in myocardial substrate metabolism contribute to myocardial dysfunction.

## Background

Heart disease is the leading cause of death in patients with type 2 diabetes mellitus (T2DM), even in the absence of coronary artery disease and hypertension, which is ascribed to diabetic cardiomyopathy (DCM) [1]. In particular, altered myocardial energy metabolism, resulting from changes in substrate supply and utilisation, has been proposed to contribute to the development of DCM [2,3]. The normal heart derives its energy mainly from oxidation of fatty acids (FA) (60–70%), glucose (30–40%) and lactate (10%) [1,4]. In contrast, T2DM is accompanied by increased lipolysis, hypertriglyceridemia, and reduced insulin-mediated myocardial glucose uptake and utilisation. This results in a shift of myocardial substrate use towards even higher FA utilisation. Reduced carbohydrate oxidation with a concomitant increase in FA oxidation and myocardial dysfunction has been shown *in vitro *in various experimental diabetic models with a severe metabolic phenotype, using isolated working hearts [5-8], whole-heart preparations [9] and ^13^C-nuclear magnetic resonance [10].

In more advanced diabetes and in the presence of compromised myocardial function, such as in heart failure and ischemia, altered myocardial substrate metabolism may further aggravate function [11]. However, there is limited knowledge regarding myocardial metabolic phenotype in relation to function in early diabetes. In the Zucker diabetic fatty (ZDF) rat, myocardial dysfunction, as determined by echocardiography, is mildly present at 14 weeks [12] and overt at 20 weeks [13], whereas these myocardial functional alterations are absent at 7 weeks of age [13]. However, these studies did not assess myocardial substrate metabolism [12,13]. Interestingly, in spite of alterations in myocardial carbohydrate metabolism, no changes in systolic function were found in 11-weeks-old ZDF rats [10]. Moreover, at 12 weeks of age, ZDF rats showed increased FA oxidation and decreased carbohydrate oxidation with only a slight depression of systolic function *in vitro *[9]. Taken together, these *in vitro *data suggest that changes in myocardial substrate metabolism may contribute to myocardial dysfunction, but *in vivo *evidence is scarce. Detailed characterisation of alterations in myocardial substrate metabolism in early diabetes *in vivo *may increase insight in the pathophysiology of myocardial dysfunction. Therefore, the purpose of the present study was to investigate the relationship between myocardial substrate metabolism and function *in vivo *in 14-weeks-old ZDF rats using state-of-the-art techniques, including [^11^C]palmitate and [^18^F]-2-fluoro-2-deoxy-D-glucose (^18^FDG) positron emission tomography (PET) under controlled conditions, together with *in vivo *echocardiography and *in vitro *analysis of myocardial function. We found increased myocardial FA oxidation with a concomitant decrease of insulin-mediated myocardial glucose utilisation *in vivo*, whereby the latter was associated with impaired myocardial function in early DCM.

## Methods

### Animals

All experiments were approved by the Animal Care and Use Committee of the VU University, and were conducted in accordance with both the European Convention for the Protection of Vertebrate Animals used for Experimental and Other Scientific Purposes, and the Dutch Animal Experimentation Act.

Male ZDF (*fa/fa*; n = 16) and age-matched Zucker lean control rats (ZL; +/+; n = 12) were purchased from Charles River Laboratories (Bruxelles, Belgium) at 11 weeks of age. Rats were maintained on Teklad 2016 (Harlan, Horst, The Netherlands), consisting of 16.7 wt% protein, 4.2 wt% fat and 60.9 wt% carbohydrates, *ad libitum*. Animals were housed in a temperature-controlled room (20–23°C; 40–60% humidity) under a 12/12 h light/dark cycle starting at 6.00 am. Body weight (BW), caloric intake and water intake were determined on a weekly basis. At 14 weeks of age, animals underwent echocardiography and PET. A separate group of rats (n = 4 ZDF rats, n = 4 ZL rats) received insulin stimulation through an i.p. injection of 10 U/kg BW insulin (Actrapid 100 U/ml; Novo Nordisk, Denmark). The effects of insulin were compared with those in animals that received a saline injection (n = 4 ZDF rats, n = 4 ZL rats). After a 6 h fast and after thirty minutes of saline or insulin injection, rats were killed by decapitation. Trunk blood was collected for plasma determinations. Hearts were removed and either snap-frozen in dry-ice-chilled isopentane, liquid nitrogen or fixed in 4% formalin for further biochemical and histological analysis.

### Echocardiography

Echocardiography (ALOKA ProSound SSD 4000, Aloka, Tokyo, Japan) was performed as described previously [14]. Briefly, rats were anaesthetised with 3% isoflurane via an induction chamber, and maintenance was performed by 1.5–1.8% isoflurane and 0.6 L/min O_2 _throughout the procedure via spontaneous breathing. Heart rate (HR) was kept relatively constant throughout the procedure. All parameters were averaged over at least three cardiac contractile cycles. Wall thickness (WT) and LV-dimensions during end-systole (ESD) and end-diastole (EDD) were determined in the B-(brightness) mode of the parasternal short-axis view at the level of the papillary muscles. Left ventricular (LV) systolic function was calculated by 3 independent parameters; 1) fractional shortening (FS), which was calculated by the equation: FS (%) = (EDD-ESD)/EDD·100, 2) LV ejection time (ET), calculated from the aorta flow waveform obtained from the apical five-chamber view by measuring the interval from the beginning of acceleration to the end of deceleration, and 3) by the systolic excursion of the mitral annulus (MAPSE), a measurement for the displacement of the mitral annulus, which was measured by M-(motion) mode echocardiography of the apical four-chamber view. LV diastolic function was assessed by pulsed wave Doppler analyses. Early transmitral peak diastolic flow velocity (E-wave), deceleration time of the E-wave (Edec) and isovolumic relaxation time (IVRT) were obtained from the apical four-chamber view. Peak early (E') diastolic tissue velocity was recorded using Tissue Doppler Imaging at the lateral side of the mitral annulus. Cardiac index (CI) and LV mass index (LVMI) were determined as described previously [14]. Analyses were performed off-line (Image-Arena 2.9.1, TomTec Imaging Systems, Unterschleissheim/Munich, Germany).

### Cannulation of the jugular vein and femoral artery

Directly after echocardiography, catheters were placed in the jugular vein and femoral artery under Hypnorm (0.5 ml/kg; Janssen Pharmaceutics, Beerse, Belgium; fentanyl citrate 0.315 mg/ml, fluanisone 10 mg/ml) and Dormicum (0.8 ml/kg; Midazolam, Roche, Woerden, The Netherlands) anaesthesia. Insulin, glucose and PET tracers were administered via the jugular vein, and the femoral artery was used for blood sampling. After surgery, rats were allowed to recover for a period of 3–5 days.

### Myocardial positron emission tomography

After a 10 h fast, anaesthesia was induced using 3.5–4.0% isoflurane, and maintained throughout the entire scanning procedure using 2.0–3.0% isoflurane and 1.0 L/min via spontaneous breathing. PET studies were performed on a high resolution research tomograph (HRRT; CTI/Siemens, Knoxville, TN) with an intrinsic spatial resolution of <3.0 mm full width at half maximum over the whole field of view [15]. For measurement of myocardial FA metabolism, 15.3 ± 0.6 MBq of [^11^C]palmitate was injected and flushed with 0.2 ml citrate buffer (0.01 mM) followed by a 1 h emission scan. After a 3 h wait to allow for radioactive decay of ^11^C, 13.4 ± 0.7 MBq of ^18^FDG was injected followed by a 1 h emission scan for measurement of myocardial glucose metabolism. This scan was performed during the steady-state of a hyperinsulinaemic euglycaemic clamp. For both tracers, data were sorted into 21 frames (1 × 30, 7 × 10, 1 × 20, 2 × 30, 2 × 60, 2 × 150, 2 × 300 and 4 × 600s). Prior to both scans, a 6 min transmission scan was acquired in order to correct for tissue attenuation. After completion of the PET study, animals were killed by decapitation under isoflurane anaesthesia.

### Hyperinsulinaemic euglycaemic clamp

The hyperinsulinaemic euglycaemic clamp was initiated by 3 minutes insulin priming at an infusion rate of 120 mU/kg/min (Actrapid Penfill^®^, Novo Nordisk, Denmark), followed by constant infusion of insulin at a rate of 12 mU/kg/min. Blood glucose (BG) concentrations were determined every 5 minutes. Simultaneously, a 20% glucose solution was infused at a variable rate to maintain euglycaemia (BG 5.5 ± 0.1 mmol/L). Glucose clearance (*M*-value, mg/kg/min) was calculated as the average glucose infusion rate during the last 30 minutes.

### Analysis of PET data

**Figure 1:**
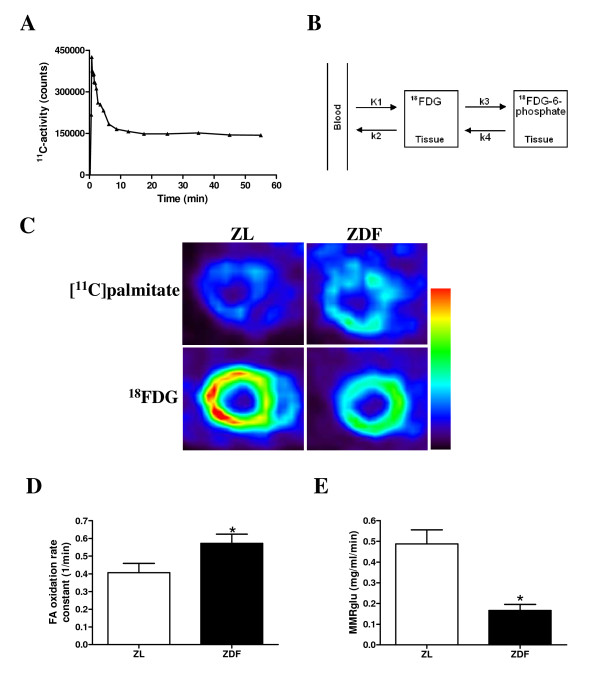
***In vivo *alterations in myocardial substrate metabolism**. Myocardial [^11^C]palmitate time-activity curve, where the rapid decline reflects clearance of palmitate from the myocardium (**A**). Two-tissue compartment model used for determining myocardial metabolic rate of glucose utilisation (MMRglu) (**B**). Typical example of a myocardial [^11^C]palmitate and ^18^FDG image in a ZL and ZDF rat normalised for standard uptake value (summed images from 30 to 60 min) (**C**). Myocardial fatty acid (FA) oxidation rate constant measured under fasting conditions (**D**), and MMRglu measured under hyperinsulinaemic euglycaemic clamp conditions (**E**) for ZL rats (open bars) and ZDF rats (filled bars). Data are expressed as mean ± SEM, n = 6–11, * p < 0.05 vs. ZL rats.

PET data were reconstructed using a partial volume corrected – ordered subset expectation maximisation (PVC-OSEM) algorithm [16], leading to an expected effective spatial resolution in the images of ~2.0 mm. Reconstructed images consisted of 256 × 256 × 207 voxels, whereby one voxel measures 1.2 mm in each direction. Regions of interest (ROIs) were placed over the LV cavity and the myocardium in three image planes and decay corrected time-activity curves (TACs) were obtained (AMIDE 0.8.22, amide.sourceforge.net). Regions near the liver were avoided to prevent spillover to the heart. Myocardial [^11^C]palmitate oxidation rate was derived from a biexponential fit to the TAC (Figure 1A), excluding data points measured before the myocardial uptake reached a maximum. The myocardial oxidation rate equals ln(2) times the inverse of the time constant of the fast exponential [17]. Quantification of myocardial ^18^FDG utilisation was achieved with compartment analysis, based on a two-tissue compartment model (Figure 1B). The input function was derived from a ROI drawn in three consecutive mid-ventricular slices, with 4 voxels per plane as centrally placed in the cavity as possible. A constant factor of 1.6 was used for the activity concentration ratio between whole blood and plasma. The value of Ki, which represents the fractional rate of tracer influx, was obtained using: Ki = K_1_·k_3_/(k_2_+k_3_), where K_1 _and k_2 _are rate constants for forward and reverse capillary transport of ^18^FDG and k_3 _refers to the rate of phosphorylation of ^18^FDG, as illustrated in figure 1B. The myocardial metabolic rate of glucose utilisation (MMRglu) is then given by MMRglu = Ki·C_glu_, where Cglu is the BG concentration.

### Force measurements in isolated cardiomyocytes

**Figure 2:**
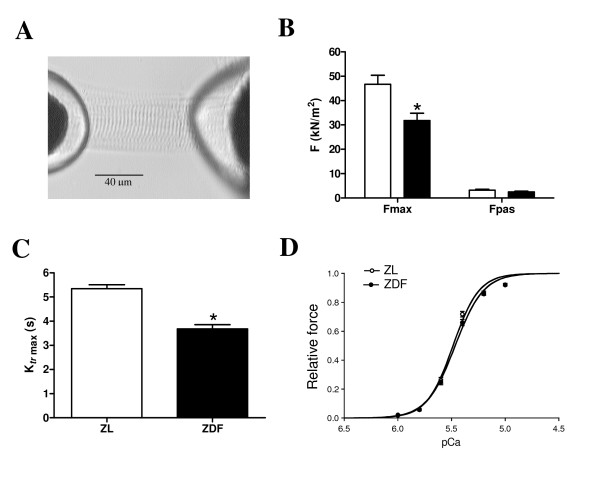
***In vitro *function of single cardiomyocytes**. Single cardiomyocyte mounted between a force transducer and piezoelectric motor (**A**). Maximal (F_max_) and passive (F_passive_) force (**B**), maximal rate of force redevelopment (K_*tr *_max) (**C**) and calcium sensitivity (**D**) in ZL rats (open bars) and ZDF rats (filled bars). Data are expressed as mean ± SEM, n = 4, * p < 0.005.

Force measurements were performed in single, mechanically isolated cardiomyocytes as described previously [18]. Briefly, LV samples (frozen after saline injection) were defrosted in relaxing solution (free Mg 1, KCl 100, EGTA 2, Mg-ATP 4 and imidazole 10 mmol/L; pH 7.0), mechanically disrupted and incubated for 5 minutes in relaxing solution supplemented with 0.5% Triton X-100 to remove all membrane structures. Subsequently, cells were washed twice in relaxing solution. Thereafter, a single cardiomyocyte was attached between a force transducer and a piezoelectric motor (Figure 2A). Resting sarcomere length of isolated cardiomyocytes was 1.8 μm and was adjusted to 2.2 μm for measurements of isometric force. The pCa (-10log [Ca^2+^]) ranged from 9.0 (relaxation solution) to 4.5 (maximal activation). All force values were normalised for cardiomyocyte cross-sectional area. The cardiomyocyte was transferred from relaxing to activating solution and the isometric force started to develop. Once a steady-state force level was reached, the cell was shortened to 80% of its original length within a period of 1 ms to determine the base line of the force transducer. The distance between baseline and steady-state force level is the total force (F_total_). After 20 ms the cell was stretched again and returned to the relaxing solution, in which a second slack-test of 10 seconds duration was performed to determine passive force (F_passive_). The difference between F_total _and F_passive _is the maximal force (F_max_) developed by the cardiomyocyte. Rate of force redevelopment was determined at maximal activation (K_*tr *_max) using the slack-test as described previously [19].

### Plasma measurements

Plasma hematocrit levels were determined using microcentrifugation. BG levels (Precision Xceed Blood Glucose monitoring system, MediSense, UK), plasma insulin (LINCO research, St. Charles, Missouri), plasma FA (WAKO NEFA-C, Wako Pure Chemical Industries, Osaka, Japan) and plasma triglyceride (TG; Sigma, Saint Louis, Missouri) levels were measured from trunk blood as described previously [14,20].

### Real-time PCR

Total RNA was extracted from 30 mg frozen LV tissue (saline injected rats). After proteinase-K treatment and DNase-digestion, RNA was purified using RNeasy mini colums (Qiagen, Venlo, The Netherlands) as previously described [21]. A total of 1 μg RNA was transcribed into complementary DNA using a superscript™ first strand synthesis kit (Invitrogen, Breda, The Netherlands) using oligo-dT priming. mRNA abundance was measured using a StepOne Plus real-time PCR system (Applied Biosystems, Carlsbad, CA). *Glucose transporter-4 *(*GLUT4*) rat specific primer pairs were designed using Primer Express software (version 3.0; Applied Biosystems, Carlsbad, CA) and are listed in the additional file 1. Reactions were carried out as previously described [21]. All mRNA expression levels were expressed relative to EF1a and HPRT abundance.

### Protein analysis

LV biopsies were homogenised to obtain cellular protein fractions for western blot analysis as previously described [14]. Phosphorylation and/or expression of signaling intermediates were analysed using the following primary antibodies: phospho-Akt-Ser473, AMP activated protein kinase α1/2 (AMPKα1/2), phospho-AMPKα1/2-Thr172 (all Cell signaling Technology, Beverly, MA), pyruvate dehydrogenase kinase-4 (PDK4; Santa Cruz Biotechnology, Santa Cruz, CA), Akt2, phospho-phospholamban-Ser16 (phospho-PLB-Ser16) (both Upstate, Lake Placid, NY), Glucose transporter 4 (GLUT4; Abcam, Cambridge, MA) and FA transporter (FAT)/CD36 (MO25) [14]. Sarcoplasmic reticulum calcium ATPase 2a (SERCA2a) was kindly provided by Dr. W. Simonides (VU University Medical centre, Amsterdam, The Netherlands). All signals were normalised to actin (Sigma, Saint Louis, Missouri). Immunoblots were quantified by densitometric analysis of films (AIDA, 4.21.033, Raytest, Strau-benhardt Germany).

### Histology

LV tissue from the free wall at the level of the papillary muscles was fixed in 4% formalin and embedded in paraffin. Sections (4 μm) were cut in the direction of the fibers, and mounted on 3-aminopropyltriethoxisilane-coated slides (Superfrost^® ^Plus, Menzal, Darmstadt, Germany). After deparaffinisation and rehydration, immunohistochemical staining and determination of the of the abundance of FAT/CD36 at the sarcolemma was performed as described before [14].

### Statistical analysis

All data are presented as mean ± SEM. Between group comparisons were performed using Student t-test. Two-way ANOVA with Bonferroni post-hoc analysis was used for group comparisons. Correlations were calculated by the Pearson's test. p < 0.05 was considered as significant.

## Results

### T2DM phenotype

ZDF rats showed increased caloric intake and BW in comparison with age matched ZL controls (Table 1). Furthermore, ZDF rats were hyperglycaemic as reflected by increased BG levels and water intake relative to ZL controls. No differences were found in hematocrit levels between animal groups. Plasma levels of insulin and TG were increased 6- and 4-fold, respectively, in ZDF vs. ZL rats, while plasma FA levels were similar in both groups. Steady-state clamp BG levels were comparable in both groups (5.6 ± 0.1 and 5.4 ± 0.1 mmol/L for ZDF and ZL groups, respectively), whereas the *M*-value was significantly lower in ZDF vs. ZL rats (Table 1).

**Table 1:** Characteristics of ZL (+/+) and ZDF (*fa/fa*) rats at 14 weeks of age

	ZL (+/+)	ZDF (*fa/fa*)
Caloric intake [kcal/wk/100 grBW]	149 ± 5	229 ± 12*
Body weight at killing [g]	296 ± 5	349 ± 4*
Water intake [ml/wk/100 grBW]	65 ± 5	264 ± 17*
Non-fasting BG [mmol/L]	5.4 ± 0.2	20.1 ± 1.0*
Hematocrit [%]	49.7 ± 1.6	54.4 ± 1.7
Fasting insulin [pmol/L]	66.0 ± 5.8	387.8 ± 74.4*
Fasting TG [mmol/L]	0.58 ± 0.07	2.11 ± 0.54*
Fasting FA [mmol/L]	0.62 ± 0.05	0.58 ± 0.05
*M*-value [mg/kg/min]	21.2 ± 1.2	8.4 ± 0.7 *

Data are presented as mean ± SEM, n = 8–10, * p < 0.05 vs. ZL rats.

ZL, Zucker lean; ZDF, Zucker diabetic fatty; BG, blood glucose; TG, triglyceride; FA, fatty acid.

### Myocardial function in vivo and in vitro

*In vivo *B-mode echocardiography revealed increased LV lumen diameter and reduced wall thickness during systole in ZDF versus ZL rats (Table 2). LVMI was comparable in both groups. Systolic functional abnormalities in ZDF relative to ZL rats were demonstrated by 3 independent parameters, i.e. reduced FS, MAPSE, and prolonged ET (Table 2). No differences were found in CI. ZDF vs. ZL rats showed prolonged IVRT, E deceleration time, decreased E-wave and decreased E', collectively indicating impaired LV relaxation and filling (Table 2).

**Table 2:** Echocardiographic parameters of ZL (+/+) and ZDF (*fa/fa*) rats

	ZL (+/+)	ZDF (*fa/fa*)
Heart rate [bpm]	303 ± 13	301 ± 7
LV mass index [mg/g]	3.0 ± 0.14	2.8 ± 0.11
**LV dimensions**		
ED lumen diameter [mm]	7.8 ± 0.2	7.9 ± 0.1
ES lumen diameter [mm]	3.2 ± 0.1	4.3 ± 0.1*
Diastolic WT [mm]	1.6 ± 0.03	1.7 ± 0.04
Systolic WT [mm]	3.3 ± 0.07	2.8 ± 0.06*
ED diameter [mm]	11.0 ± 0.2	11.1 ± 0.1
ES diameter [mm]	9.6 ± 0.4	10.2 ± 0.1
**Systolic parameters**		
Fractional shortening [%]	58.7 ± 1.3	44.8 ± 1.2*
Ejection time [ms]	82.3 ± 1.8	89.9 ± 1.6*
MAPSE [mm]	2.1 ± 0.09	1.5 ± 0.06*
Cardiac index [ml/min/g]	0.29 ± 0.02	0.27 ± 0.02
**Diastolic parameters**		
IVRT [ms]	26.7 ± 2.3	39.6 ± 2.3*
E deceleration time [ms]	38.4 ± 2.2	54.4 ± 3.0*
E wave [cm/s]	116.6 ± 4.2	98.7 ± 2.3*
E' [cm/s]	9.4 ± 0.3	8.4 ± 0.4^‡^

Data are presented as mean ± SEM, n = 12, * p < 0.01 and ^‡ ^*P *= 0.07 vs. ZL rats.

ZL, Zucker lean; ZDF, Zucker diabetic fatty; LV, left ventricle; ED, end-diastolic; ES, end-systolic; WT, wall thickness; MAPSE, mitral annulus plane systolic excursion; IVRT, isovolumic relaxation time.

*In vitro *single cardiomyocytes from ZDF vs. ZL rats showed significant decreases in F_max _(Figure 2B) and k_*tr *_max (Figure 2C), whereas both groups showed comparable F_pas _(Figure 2B) and calcium sensitivity of the myofilaments (Figure 2D).

### Myocardial substrate metabolism

Figure 1C shows typical myocardial [^11^C]palmitate and ^18^FDG images (summed images from 30 to 60 min) in a ZDF and ZL rat. Myocardial FA oxidation rate was significantly increased in ZDF animals as compared to ZL rats (Figure 1D), whereas MMRglu was significantly lower in ZDF versus ZL animals (Figure 1E).

### Molecular alterations in ZDF versus ZL rats

**Figure 3:**
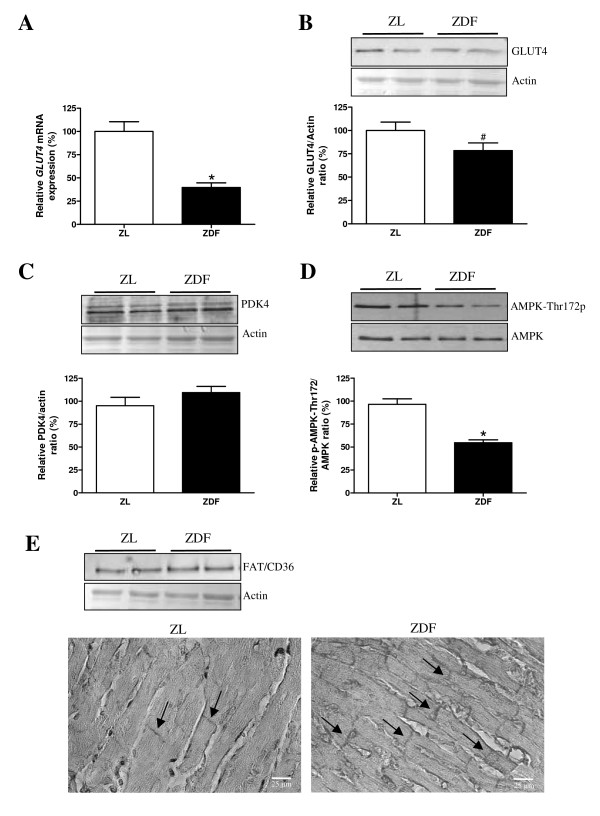
**Molecular alterations in ZDF hearts**. Quantification of *glucose transporter-4 *(*GLUT4*) mRNA levels (**A**) and protein levels (**B**), pyruvate dehydrogenase 4 (PDK4) protein levels (**C**) and phosphorylation/total protein levels of AMP kinase a (AMPKα1/2-Thr172) (**D**) in ZL rats (open bars) and ZDF rats (filled bars). Protein expression and a typical example of subcellular localisation of the fatty acid transporter (FAT)/CD36 (**E**) in ZL rats and ZDF rats. Data are expressed as mean ± SEM, n = 4–8, * p < 0.05, ^# ^p = 0.10.

*GLUT4 *mRNA and protein expression were reduced by 68% (p < 0.05) and 22% (p = 0.10), respectively, in ZDF versus ZL hearts (Figure 3A and 3B). PDK4 was comparable in both groups (Figure 3C), whereas phosphorylation/total protein ratio of AMPKα1/2 was significantly decreased in ZDF vs. ZL hearts (Figure 3D). In the absence of changes in total expression levels, histological analyses (Figure 3E) showed a 2.4-fold increase (p < 0.01) in the sarcolemmal abundance of FAT/CD36 in ZDF hearts (31.0 ± 2.5%) as compared to ZL hearts (12.9 ± 1.0%), indicative for increased FA uptake.

**Figure 4:**
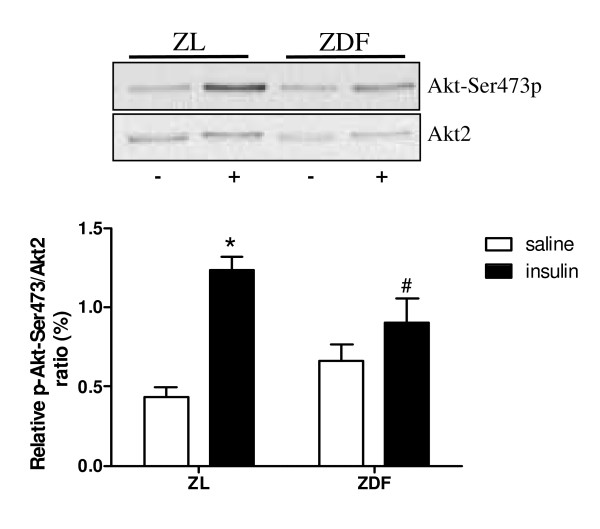
**Molecular alterations in myocardial insulin signaling**. Quantification of immunoblots showing relative phosphorylation/total protein levels of Akt-Ser473/Akt2 after saline (-; open bars) or insulin (+; filled bars) injection. Data are expressed as mean ± SEM, n = 4–8, * p < 0.05 different from basal, # p < 0.05 different from ZL.

ZDF versus ZL hearts showed a significant decrease in myocardial insulin-mediated Akt-Ser473 phosphorylation (Figure 4), whereas no differences were found in basal phosphorylation. Finally, intracellular signaling molecules related to myocardial calcium handling, like PLB and SERCA2a were studied. No difference was found in SERCA2a protein expression (see additional file 2A). Basal phosphorylation of PLB-ser16, however, was decreased in ZDF compared to ZL hearts, although it failed to reach significance (p = 0.08, see additional file 2B).

### Association between myocardial metabolism and function

**Figure 5:**
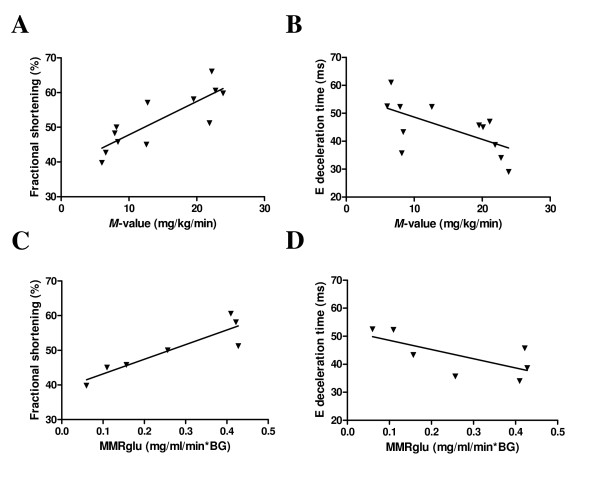
**Association between metabolism and function**. Relationship of whole-body insulin sensitivity (*M*-value) with fractional shortening (**A**; r = 0.83, p < 0.001, n = 12) and E deceleration time (**B**; r = -0.62, p < 0.05, n = 12). Relationship of myocardial metabolic rate of glucose utilisation (MMRglu) with fractional shortening (**C**; r = 0.91, p < 0.005, n = 7) and E deceleration time (**D**; r = -0.70, p = 0.08, n = 7).

Whole-body insulin sensitivity was significantly associated with *in vivo *fractional shortening, a measure for systolic function (r = 0.83, p < 0.001; Figure 5A) and inversely related to E deceleration time, a measure for diastolic function (r = -0.62, p < 0.05; Figure 5B). No association was found between FA oxidation and myocardial function. In contrast, MMRglu was strongly associated with fractional shortening (r = 0.91, p < 0.005; Figure 5C) and showed a trend to be inversely associated with E deceleration time (r = -0.70, p = 0.08; Figure 5D).

## Discussion

This study demonstrates alterations in myocardial substrate metabolism and impaired myocardial function in a rat model of early DCM. In particular, using *in vivo *PET and echocardiography, our data extend previous *in vitro *studies that assessed function [12,13], metabolism [22] or both [9,10]. In particular, the present data showed increased myocardial FA oxidation with a concomitant decrease in insulin-mediated myocardial glucose utilisation. In addition, changes in myocardial function were associated with systemic insulin sensitivity and reduced myocardial glucose utilisation, whereas no relation was found between function and myocardial FA oxidation.

Emerging evidence supports the concept that alterations in myocardial substrate metabolism contribute to myocardial dysfunction. In this regard it is conceivable that alterations in substrate metabolism and underlying molecular changes precede the development of overt myocardial function. Previously, the earliest reported time point at which there was evidence of heart failure in ZDF rats was at 20 weeks of age [13], 6 weeks after the age of the rats reported here. In addition to metabolic alterations, echocardiographic diastolic and systolic functional impairment and *in vitro *myofilament dysfunction in isolated cardiomyocytes were found. Impaired myocardial carbohydrate utilisation in the absence of systolic dysfunction in 11-weeks-old ZDF rats has been observed using ^13^C-nuclear magnetic resonance [10], while at 12 weeks of age, ZDF rats have shown increased FA oxidation and decreased carbohydrate oxidation in isolated hearts with depressed systolic function [9], suggesting that changes in myocardial substrate metabolism precede myocardial dysfunction. The present *in vivo *data demonstrate that myocardial glucose utilisation, but not FA oxidation, is associated with both diastolic and systolic function. Thus, myocardial dysfunction seems closely related to the severity of altered myocardial substrate metabolism in early diabetes.

The information provided by *in vitro *studies regarding myocardial substrate metabolism in relation to myocardial function is restricted by experimental conditions. In most studies, the choice of substrate concentrations may not truly reflect what the heart utilises *in vivo*. In contrast, PET provides a means for directly measuring true substrate metabolism *in vivo*. Here, [^11^C]palmitate and ^18^FDG PET were used to assess myocardial FA and glucose metabolism under fasting and hyperinsulinaemic euglycaemic clamp conditions, respectively. Previously, [^11^C]palmitate PET was performed in 12-weeks-old ZDF rats [22], showing increased FA oxidation. In spite of a reciprocal relationship between FA en glucose utilisation in the heart [1,3,4], increased myocardial glucose utilisation measured with [^11^C]glucose was reported. In contrast, the same research group, now using ^18^FDG PET, found a decrease in myocardial glucose uptake rate and utilisation in 19-weeks-old ZDF rats [23], possibly due to the difference in age and in glucose tracer. These differences might also be explained by the conditions under which glucose utilisation was measured, i.e. under fasting conditions, resulting in FA being the primary substrate for myocardial energy. In contrast, in the present study glucose utilisation was measured using ^18^FDG under controlled hyperinsulinaemic euglycaemic conditions, as it is known that these conditions yield the best ^18^FDG image quality and the highest glucose utilisation in comparison with a glucose load or an insulin bolus [24,25]. Taking this approach, increased FA oxidation and decreased glucose utilisation were found in this early stage of DCM in the hearts of ZDF rats. In agreement with physiological data, impaired myocardial insulin sensitivity in ZDF hearts was demonstrated by an impaired ability of insulin to phosphorylate Akt. Similar results were reported in hearts from high-fat diet fed rats [20], Zucker rats [26] and ob/ob mice [27], whereby the latter showed that impaired insulin signaling is associated with alterations in myocardial glucose metabolism measured in isolated working hearts. Further, we showed that systemic insulin sensitivity, measured as whole-body insulin sensitivity (*M*-value), was significantly associated with *in vivo *systolic and diastolic function. Also, myocardial insulin sensitivity, measured as insulin-mediated myocardial glucose utilisation, correlated with systolic and diastolic function. To the best of our knowledge, this is the first report in rats showing an association between *in vivo *function and *in vivo *myocardial metabolism. The myocardium is a metabolic omnivore that under healthy conditions will rely on FA oxidation for the largest part of its ATP production. However, as glucose is the more energetically efficient substrate, the myocardium should be readily able to switch to glucose under conditions of stress (e.g. ischemia, myocardial functional impairment such as in heart failure). As insulin resistance impacts on myocardial substrate supply (e.g. by increasing triglyceride-rich lipoprotein- and glucose output from the liver and adipose tissue-derived free fatty acids through unsuppressed lipolysis), it is clear that both systemic and organ-specific impairment of insulin action will influence substrate utilisation and reduce myocardial "metabolic flexibility" [28-31]. Thus, in the stressed heart insulin resistance may finally hamper energy metabolism and as such myocardial function [28,29,31]. Conversely, improvement of insulin sensitivity may ameliorate myocardial function. In humans, Iozzo *et al*. found an inverse association of myocardial insulin-mediated glucose utilisation and systolic function [31]. Our group showed that the insulin sensitizer pioglitazone improved whole-body insulin sensitivity and insulin-mediated myocardial glucose utilisation together with an improvement in myocardial diastolic function in patients with well-controlled T2DM [29]. These findings support the role of insulin sensitivity in myocardial metabolism and function in DCM.

A restriction to glucose utilisation in the diabetic heart is the slow rate of glucose transport across the sarcolemmal membrane into the myocardium [32]. In line with previous reports [6,33], decreased GLUT4 expression was found. PDK4 has an inhibitory effect on glucose oxidation via the pyruvate dehydrogenase complex (PDH) [34], however, no differences were found in PDK4 protein expression. Similar PDK4 mRNA levels were found in ZDF rats [6] and Zucker rats [5]. However, Chatham *et al*. [10] showed increased PDH activity in ZDF hearts. AMPK is known as a major regulator of metabolic myocardial energy substrate acting as a metabolic sensor following an energetic imbalance. Decreased AMPKα1/2 phosphorylation was found, which is consistent with previous results [35,36]. In addition, a significant increase in sarcolemmal localisation of the FA transporter FAT/CD36 in ZDF hearts was seen, which is compatible with the observed enhancement of myocardial FA oxidation. These data are in line with those reported in high-fat diet fed [14] and Zucker [37,38] rats, in both of which relocation of FAT/CD36 to the sarcolemmal membrane was associated with increased myocardial FA uptake. Collectively, these molecular findings are compatible with the measured shift in myocardial substrate metabolism towards increased myocardial FA metabolism and decreased myocardial glucose metabolism.

In isolated cardiomyocytes of ZDF rats, significant reductions were observed in both maximal force and maximal rate of force redevelopment. A previous study by Ren *et al*. [39] showed reduction in peak shortening, prolonged duration of re-lengthening and unaltered resting intracellular calcium levels in intact cardiomyocytes from 14-weeks-old Zucker rats. The reduction in maximal force generating capacity of myofilaments observed in the present study may well explain reduced cardiomyocyte shortening. Moreover, the reduction in maximal rate of force redevelopment may, at least in part, underlie prolonged duration of cellular re-lengthening (i.e. cardiomyocyte relaxation). No differences were found in calcium sensitivity as well as SERCA2a expression. However, a trend towards decreased phosphorylation of phospholamban was found. Hence, cellular dysfunction in early DCM could be the result of myofilament dysfunction. Nevertheless, the exact mechanisms leading to depressed myocardial function in early diabetes remain elusive. Several potential options to be responsible for the pathogenesis of myocardial dysfunction in early diabetes have been postulated, including (1) lipotoxicity via ceramide dependent pathways, (2) increased accumulation of advanced glycation end products, and (3) generation of reactive oxygen species via increased flux through mitochondrial pathways. Further studies are necessary to reveal the exact transition from compensatory myocardial function to overt myocardial dysfunction during alterations in myocardial substrate metabolism.

## Conclusion

Using combined *in vivo *measurements of myocardial substrate metabolism and function by state-of-the-art PET and echocardiography, respectively, we found increased myocardial FA oxidation under fasting and decreased insulin-mediated glucose utilisation under (hyperinsulinaemic) isoglycaemic conditions as well as a reduced systolic and diastolic function in early experimental DCM. Furthermore, myocardial glucose utilisation was associated with myocardial function. At the molecular level, the alterations in myocardial substrate metabolism were paralleled by a decreased expression of GLUT4 and increased sarcolemmal abundance of FAT/CD36. Finally, the observed association between impaired systemic and myocardial insulin sensitivity and myocardial dysfunction supports the notion that alterations in myocardial substrate supply and metabolism may affect the course of DCM.

## Competing interests

The authors declare that they have no competing interests.

## Authors' contributions

CEvdB participated in performing the study, data analysis, statistics and writing the manuscript. MCH in part performed data analysis. RV in part performed the study and contributed to writing the manuscript. NMB and SD in part performed the study. ML in part performed data analysis. CFMM in part performed the study and contributed to the design of the study. AAL in part participated in the design of the study and reviewed/edited the manuscript. JvdV reviewed/edited the manuscript. CB contributed writing and reviewed/edited the manuscript. DMO participated in the design of the study and reviewed/edited the manuscript. MD supervised the study, participated in the design of the study and wrote/reviewed/edited the manuscript. All authors read and approved the final manuscript.

## Supplementary Material

Additional file 1 **Primer list**. List of primers used Click here for file

Additional file 2 Molecular alterations in calcium handling Click here for file
