# Supplementary material for: Altered myocardial substrate metabolism is associated with myocardial dysfunction in early diabetic cardiomyopathy in rats: studies using positron emission tomography
Source: Cardiovasc Diabetol. 2009 Jul 22;8:39. doi: 10.1186/1475-2840-8-39 (PMC2722582; doi:10.1186/1475-2840-8-39)
Supplement: Additional file 1 — Primer list. List of primers used [file 1475-2840-8-39-S1.pdf]

Supplemental data: Primer list

		Forward	Reversed
GLUT4	NM_012751	5’-AGGCACCCTCACTACCCTTT-3’	5’-TTTCCTTCCCAACCATTGAG-3’
EF1 α	NM_175838	5’-GGGGACAATGTAGGCTTCAA-3’	5’-TCCATTGGTGGGTCATTTTT-3’
HPRT	NM_000194	5’-GGTCCTTTTCACCAGCAAGCT-3’	5’-TGACACTGGCAAAACAATGCA-3’
