# Supplementary material for: Altered myocardial substrate metabolism is associated with myocardial dysfunction in early diabetic cardiomyopathy in rats: studies using positron emission tomography
Source: Cardiovasc Diabetol. 2009 Jul 22;8:39. doi: 10.1186/1475-2840-8-39 (PMC2722582; doi:10.1186/1475-2840-8-39)
Supplement: Additional file 2 — Molecular alterations in calcium handling [file 1475-2840-8-39-S2.pdf]

Supplemental data Figure 2:

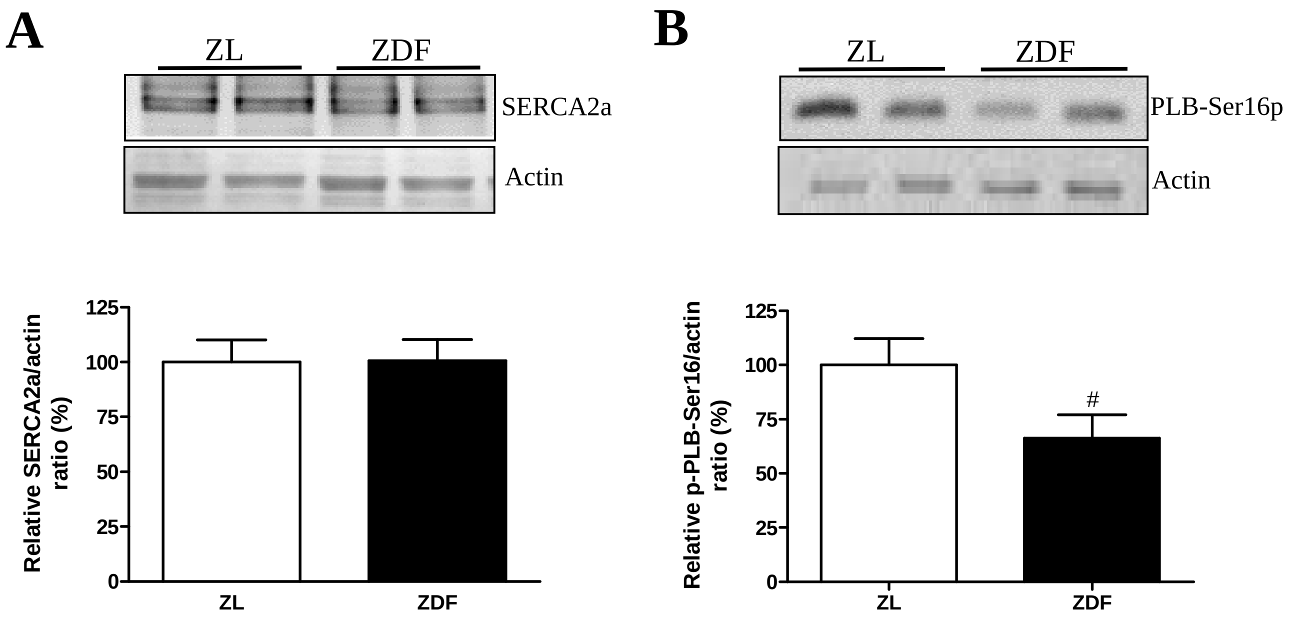

Figure 2: Molecular alterations in calcium handling

Quantification of immunoblots showing protein expression of SERCA2a (**A**), and phosphorylation of phospholamban (PLB-Ser16p) (**B**) in ZL rats (open bars) and ZDF rats (filled bars). Data are expressed as mean $\pm$ SEM, n=4-8, # p=0.08.
